# Psychological Characteristics in Talented Soccer Players – Recommendations on How to Improve Coaches’ Assessment

**DOI:** 10.3389/fpsyg.2018.00041

**Published:** 2018-02-05

**Authors:** Lisa Musculus, Babett H. Lobinger

**Affiliations:** Department of Performance Psychology, Institute of Psychology, German Sport University Cologne, Cologne, Germany

**Keywords:** diagnostics, questionnaires, sport performance, talent development, personality, expertise

## Abstract

Psychological characteristics, including personality traits and psychological skills, have been shown to be relevant predictors of soccer performance. In research, general and sport specific standardized self-report questionnaires have been applied in psychological diagnostics of sports talent. However, with regard to the assessment of psychological characteristics of talented soccer players, a gap between research and practice is apparent. While soccer clubs often ask their coaches to assess their players on self-designed, unevaluated scouting sheets, research widely neglects expert coaches’ and clubs’ perspectives on relevant performance characteristics. As we believe that expert coaches’ assessments could be a valid predictor of a player’s current performance and future success, we provide recommendations on how to improve coaches’ assessment of psychological characteristics. As the quality of the assessment of psychological characteristics is crucial, we provide recommendations on how to ensure the central diagnostic standards: objectivity, reliability, and validity in talent assessment. Further, we argue that assessing psychological characteristics should combine self ratings of players and external ratings of coaches in talent development. Sport psychologists should assist clubs and coaches in improving the diagnostics of psychological characteristics as well as in embedding psychological diagnostics and interventions in the talent development process.

## Psychological Characteristics of Talented Soccer Players

In soccer, one of the most prominent sports worldwide, coaches are part of a highly professional talent identification and development system (Mills et al., 2012; Lobinger, 2015). Within professional youth soccer academies, it is the coaches’ task to promote the physical and technical skill development as well as personality development of talented players, which reflects the belief that personality also plays a crucial role in players’ future success (Giacobbi et al., 2002). Psychological characteristics have been integrated in models of talent identification and development (e.g., the soccer-specific reviews of Morris, 2000 and Williams and Reilly, 2000) and research has identified psychological characteristics “as […] significant predictor[s] of success” in sports (Vaeyens et al., 2008, p. 706). Furthermore, in Germany, professional youth academy licensing requires clubs to focus on the psychological development of players (Lobinger et al., 2009). This is, among others, a reason why academies have started to include psychological characteristics in their general evaluation of players. For example, in recent years scouting sheets assessing technical skills (e.g., dribbling, shooting, passing) have been revised to include such psychological characteristics and youth academies ask their coaches to assess the players’ psychological characteristics regularly (Musculus and Lobinger, 2015).

Psychological characteristics within this article are seen as an umbrella term for personality traits as well as psychological skills. Personality traits are defined as a predisposition to behave in a certain way (Pervin, 1996). Psychological skills encompass structured and prepared behaviors and thinking used by athletes to control their personal psychological state (Eccles and Riley, 2014). Whereas personality traits are conceptualized as being relatively stable across time and situations (Aidman and Schofield, 2004), psychological skills are less stable, meaning that they can change according to the situation or context. Consequently, the conceptualization as trait or skill determines how often the respective characteristics should be assessed.

In psychological diagnostics of sports talent and of soccer talent, two main approaches using standardized questionnaires have emerged and been used in empirical research to assess psychological characteristics. The first approach relies on existing personality questionnaires and the second approach uses sport-specific instruments (Morris, 2000; Tenenbaum et al., 2012). The first approach has used general personality questionnaires to measure skilled soccer players’ personality (cf. Morris, 2000). In a study published more than 50 years ago, Kane (1966) administered the 16 Personality Factors Questionnaire (Cattell, 1956) to professional adult and youth players of England’s first division clubs, Cooper and Payne (1972) applied the Orientation Inventory (Bass, 1962), and Panda and Bisivas (1989) used the Eysenck Maudsley Personality Inventory (Eysenck, 1956). These early studies applying general questionnaires did not help in outlining personality profiles that differentiated between more and less successful soccer players. Although the standardized questionnaires are objective and reliable for the general population, when applied in a sports context they are not sensitive to the homogenous group of highly skilled athletes or players (Morris, 2000; Höner and Feichtinger, 2016). A lack of validity of general personality questionnaires is one of the reasons why the second approach emerged.

In this vein, specific questionnaires and methods for assessing psychological characteristics relevant for sports were developed (Morris, 2000; Elbe et al., 2003), referred to as the second approach. Applying sport-specific self-report questionnaires revealed that many different psychological characteristics have been assessed in soccer, ranging from motivational aspects (see e.g., Figueiredo et al., 2009) to self-regulation (see e.g., Toering et al., 2009) or the use of coping strategies (see e.g., van Yperen, 2009). A recent review including 43 studies (14,977 participants) came to the conclusion that self-regulation, resilience, commitment, and discipline had the greatest impact on player development (Gledhill et al., 2017). Another large-scale research project conducted with 2,677 U-12 players in the German talent development program (Feichtinger and Höner, 2014; Höner and Feichtinger, 2016) assessed psychological characteristics from areas of motivation, volition, self-referential cognitions, and emotion using an online questionnaire. Players selected to play in regional association representative teams had more functional scores in the respective psychological characteristics than the less successful competence center players. Furthermore, psychological characteristics were positively associated with current and future soccer performance. While these relations can be interpreted as concurrent and predictive validity, the effect sizes in both of the above mentioned studies were reported to be small. Thus, the psychological characteristics assessed with the standardized questionnaires and the results obtained so far should not provide the base for selecting or deselecting players in terms of talent identification (Feichtinger and Höner, 2014; Höner and Feichtinger, 2016).

We conclude that the concurrent and predictive validity of the assessment of psychological characteristics can still be improved. Additionally, study results often lack clear implications for talent identification and development that can be easily implemented in the field (Abbott and Collins, 2004), yielding a gap between research and practice (Figueiredo et al., 2014). This gap is apparent in the assessment of psychological characteristics of talented players in professional youth soccer. On the one hand, clubs ask their coaches to assess their players on self-designed, unevaluated scouting sheets (Musculus and Lobinger, 2015). On the other hand, research predominantly focuses on players’ self-report. Indeed, expert coaches’ and clubs’ perspectives on important performance characteristics have mostly been neglected in research so far (Mills et al., 2012; Huijgen et al., 2014; Gledhill et al., 2017). The review by Gledhill et al. (2017) revealed that in the studies examining psychological characteristics less than 1% of the participants were coaches.

Importantly, qualitative interview studies involving coaches point out other, additional relevant psychological characteristics like awareness, passion, professional attitude (Mills et al., 2012) or conforming dedication and willingness to sacrifice (Holt and Dunn, 2004) that are not necessarily considered in standardized self-report questionnaires. This indicates that considering the coaches’ perspective provides additional insight on relevant psychological characteristics. Previous research has suggested that expert coaches’ assessments are grounded in their varied and lengthy experiences working with different players (Jones et al., 2013). Thereby, expert coaches can base their assessments of psychological characteristics on a representative sample of talented players they have worked with in the past, who differed in their personality, skills, and level of success. Coaches are able to make inter-individual comparisons and use this knowledge to evaluate and predict a player’s current and future potential. Furthermore, coaches’ and players’ evaluation of the players’ performance have been shown to differ. Players as compared to coaches overestimated their performance (van Yperen and Duda, 1999). Thus, we believe that expert coaches’ assessments can add explanatory power to the self-report of players and be a valid predictor of talented players’ future success. To improve coaches’ assessment of psychological characteristics, in the following section we provide recommendations on how to integrate coaches’ expertise and how to design diagnostically sound instruments that can be used in talent diagnostic.

## Recommendations on How to Improve the Assessment of Psychological Characteristics

Involving expert coaches in the identification and assessment of psychological characteristics that they regard as helpful in achieving outstanding performance might be a promising add-on to the two approaches presented above. In particular, the characteristics identified by expert coaches’ intuition might be helpful in characterizing talents and valid for predicting current performance and future success in soccer players (van Yperen and Duda, 1999; Williams and Reilly, 2000). The diagnostic quality of the coaches’ assessment of psychological characteristics is important not only for the individual player but also for the club investing in talent development.

To provide a sound diagnostic of psychological skills of talented soccer players, coaches’ ratings should fulfill the central diagnostic standards. Ratings should be as objective as possible, meaning preferably made independent of the individual who assesses psychological characteristics (Favor, 2011; Tenenbaum et al., 2012), such as a scout or the coach. Second, the measurement should be reliable, meaning that the characteristics should be assessed adequately and exactly with little measurement error (Tenenbaum et al., 2012). Regarding measurement validity, both concurrent and predictive validity are highly relevant. A measurement is concurrently valid if a relationship between a variable and an external criterion measured simultaneously exists (Cohen et al., 2013). An assessment is predictively valid if external criteria can be predicted based on factors assessed earlier in time (Tenenbaum et al., 2012). To optimize coaches’ assessments, several aspects with regards to objectivity, reliability, and validity of the instruments applied have to be considered.

### Improving Objectivity

As an important basis, the scouting sheets, which serve as the instruments for coaches’ assessments, should contain a clear definition of the characteristics (Musculus and Lobinger, 2015). A definition is a basis for an objective measurement because it ensures all raters share a common understanding of the objective. Therefore, we recommend definitions of the characteristics on the evaluation sheets that should be precisely elaborated in discussions with coaches, and formulated referring to observable behavior. Sport psychologists could help work out behavioral anchors that represent more general or abstract psychological characteristics being assessed (Abbott and Collins, 2004; Cohen et al., 2013). Relating the theoretical constructs to concrete behavior is not only important for assessing the psychological characteristics objectively, but might, in our opinion, also be useful during goal setting and feedback talks when coaches point out strengths and skill training for the players.

Another point that directly relates to the definition of the characteristics involves the setting of the scale. Appropriate answering formats from standardized sports-related questionnaires formats should be considered (Nau, 2011). To support objective assessment, these aspects should be integrated into a standardized evaluation sheet. A standardized evaluation sheet should include clear instructions for the coaches and short definitions of the constructs that should be evaluated on a predefined rating scale containing behavioral anchors. The importance of a standardized procedure favoring objectivity should be stressed, as it is the basis for reliability and validity.

### Improving Reliability

If an assessment is reliable, the characteristics will be assessed as precisely as possible and contain only small measurement error (Coaley, 2014). One way to achieve a higher reliability relates to the number of items the assessment is based on (Coaley, 2014). This positive relation between the number of items and reliability reflects a higher true score in relation to error variance with an increasing number of items (Coaley, 2014). From an untrained assessor‘s point of view, a psychological characteristic can perhaps be assessed by one overall score that represents the evaluation and weighting of different aspects. Which aspects of a construct should be differentiated is a question of validity, while how these aspects should be assessed is mainly a question of reliability. From a methodological perspective, we highly recommend to use more than one item to operationalize any psychological construct, and every aspect of a construct should be assessed separately by an individual item. Furthermore, the homogenous sample of talented soccer players in professional youth academies also has to be taken into account (Höner and Feichtinger, 2016). To allow for differentiation between players, the item characteristics have to be considered carefully and the construction of psychological items corresponding to different difficulty and discrimination indices should be the goal (Coaley, 2014).

To check the systematic variance, namely the true variance that reflects the construct, accounted for by an assessment, more than one rater should evaluate the same player (Cohen et al., 2013; Coaley, 2014). Therefore, we recommend at least two independent raters; the coach, the assistant coach or any other personnel, who know the player, are all reasonable choices. It is important that the raters really assess the player independently, meaning that they do not talk about the assessment beforehand and do not influence one another’s ratings. This allows quantifying reliability in terms of an inter-rater agreement, namely inter-rater reliability as a measure of agreement between multiple raters. The sport psychologist could offer support in data analysis, for example, setting up an automatized syntax to calculate inter-rater reliability and reporting results to the raters, ensuring that reasonable interpretations and implications are drawn from it (Wylleman et al., 2009; Nau, 2011). The results might, in the long run, serve as a basis for a discussion among coaches regarding the players’ development. Inter-rater reliabilities between coaches and players could provide a starting point for feedback talks and might motivate players to focus on and to further develop their psychological characteristics.

The last recommendation concerns the frequency of assessing the psychological characteristics to ensure reliability. If a coach evaluates the same player more than once, a test–retest reliability can be calculated. As traits are expected to be stable in time and skills are expected to change across situations, different test–retest scores would be expected within the same time-frame (Aidman and Schofield, 2004). While it might be sufficient to test personality traits once a year, we would suggest assessing psychological skills more often and in different settings, for example, during preseason preparation and throughout the competitive season. As this consideration links the stability to the conceptualization of psychological characteristic assessed, this is also a question of (construct) validity (Coaley, 2014).

### Improving Validity

To assess psychological characteristics in a valid way, several aspects are important. First, coaches’ expertise and intuitive beliefs should be considered when determining which psychological characteristics are relevant for current and future performance and success in soccer (Helsen et al., 2000; Cohen et al., 2013). In a next step, a conceptually and theoretically sound definition of a psychological characteristic is needed (Jeserich, 1986; Fisseni, 2004). In our opinion, sport psychologists could support this because they have the scientific background and theory-based knowledge (Wylleman et al., 2009), which is also relevant for the methodological recommendations we propose. With regards to validity, coaches should also be integrated in the construction of items (Jeserich, 1986). In particular, they could check whether the items encompass all aspects they regard as important, whether the items are coherent and whether they can be related to players’ behavior.

Related to the theoretical conceptualization, it is also important to check whether the psychological characteristics assessed have been shown to be related to criteria of interest (Cohen et al., 2013). In talent development, performance or success in soccer can be defined as the standard against which a test score is evaluated (Ford and Williams, 2012; Feichtinger and Höner, 2014; Höner and Feichtinger, 2016). Testing concurrent and predictive validity requires stakeholders to, first, define relevant performance criteria, and, second, to test the validity of the instrument used (Jeserich, 1986). To test concurrent validity, a player’s motivation to engage in a specific game and her/his game performance statistics (e.g., playing time granted, pass accuracy) assessed at the same time could be correlated. To test predictive validity, an external criteria, such as receiving a professional contract or the league in which a player plays (Ford and Williams, 2012), could be predicted by the data that had been assessed. Given the complexity and the timeframes needed to test the validity of the psychological characteristics assessed, the validation process should be systematically developed and designed.

## Conclusion

As coaches are experts and have an intuitive understanding of valid psychological characteristics, we highly recommend the inclusion of coaches’ external ratings in addition to standardized self-report questionnaires. We see great potential in considering coaches’ external ratings of psychological characteristics of players for talent development and therefore provided recommendations on how to ensure a diagnostically sound assessment within the talent development process (Lobinger, 2015). Within this perspective, we focused on the assessment of psychological characteristics as predictors of current and future soccer performance. Nevertheless, beyond performance enhancement there is much more to an ethical, holistic talent development (Mills et al., 2012; Gledhill et al., 2017; Mann et al., 2017). We agree that in talent development, psychological characteristics may also be important for promoting holistic athlete development to include players’ well-being, the formation of positive relationships, and non-sporting enhancements (Mills et al., 2012).

With respect to the assessment of psychological characteristics for talent development, we recommend combining different sources of information like the players’ self-assessment and the coaches’ external ratings (Hackfort and Birkner, 2003). Integrating self- and external ratings can be a first step in developing a multitrait-multimethod approach (Campbell and Fiske, 1959; Giacobbi et al., 2005). A multitrait-multimethod approach for psychological characteristics in talent identification and development involves the assessment of different psychological characteristics by applying a variety of methods. For example, for measuring motivation one could use a standardized questionnaire (e.g., Achievement Motives Scale by Wenhold et al., 2009), ask coaches and players to fill out standardized evaluation sheets with behavioral anchors and furthermore add interviews, behavioral observations (e.g., video analyses) or use small-sided games (Mann et al., 2017). Applying the same approach to different psychological characteristics and relating the respective measures, would also allow inferring construct validity of the instruments used for assessing psychological characteristics (Campbell and Fiske, 1959; Coaley, 2014).

To successfully implement the diagnostic recommendations provided, we believe that additional support should be provided to clubs, youth academies and coaches. On the level of sport structure, coach education programs could embed diagnostics within the standard curriculum. In detail, providing insight into the basic diagnostic principles and quality criteria might demonstrate the importance of a sound assessment and help to close the gap between research and practice (Figueiredo et al., 2014). On the level of human resources, in line with licensing requirements (Lobinger et al., 2009), we encourage a cooperation with sport psychologists. Indeed, a lot of soccer academies have hired sport psychologists to support the players and coaches in implementing the goals set for their players’ personality and well-being as well as psychological skill development.We argue that sport psychologists should not only offer psychological interventions but also support diagnostics of psychological characteristics in talented athletes. Licensed sport psychologists are trained in theory-based diagnostics, and assessment can be considered one of the core services psychology can provide for the applied field of sport (Wylleman et al., 2009). First, sport psychologists can help in relating relevant characteristics to theory-based psychological constructs and ensure a diagnostically sound measurement of it. Second, sport psychologists can support coaches’ assessments by providing feedback on the process and evaluating results. In the long run, applying a multitrait-multimethod approach and repeated measurements can provide the basis for an integrated, individual, long term development plan for the players. Determined by diagnostics and through theory-based interventions, sport psychologists should help developing the players’ sport psychological skills according to their individual needs (Birrer and Morgan, 2010).

Educating and supporting coaches in psychological diagnostics and cooperating with sport psychologists could promote higher quality standards with respect to psychological diagnostics in talent development programs. Considering the presented recommendations on how to improve objectivity, reliability, and validity of psychological assessments in addition, could be seen as an internal quality-management procedure for professional youth academies (Lobinger and Musculus, 2011). Consequently, improved coaches’ assessments of psychological characteristics of talented players can, in turn, result in better predictions of (future) performance, which is beneficial for all stakeholders in talent development.

## Author Contributions

## Conflict of Interest Statement

The authors declare that the research was conducted in the absence of any commercial or financial relationships that could be construed as a potential conflict of interest.
